# The immunothrombosis pathway linking pulmonary disease to stroke and neurodegeneration: mechanisms and therapeutic implications

**DOI:** 10.3389/fphar.2026.1811794

**Published:** 2026-03-31

**Authors:** Aiping Zou, Huimin You, Jian Xiao

**Affiliations:** The First People’s Hospital of Jiashan, Jiashan Hospital Affiliated to Jiaxing University, Jiaxing, Zhejiang, China

**Keywords:** immunothrombosis, pulmonary disease, stroke, neurodegeneration, thromboinflammation

## Abstract

Pulmonary diseases increasingly reshape vascular biology and coagulation beyond the lung. Across acute infections and acute respiratory distress syndrome (ARDS), chronic airway inflammation (e.g., COPD), sleep-disordered breathing, fibrotic interstitial lung disease, and particulate air pollution, convergent immune programs couple inflammation to coagulation through immunothrombosis. Physiologic immunothrombosis can confine pathogens within the microvasculature, but dysregulated thromboinflammation drives endotheliopathy, platelet–leukocyte cooperation, neutrophil extracellular trap (NET) formation, complement activation, tissue factor–thrombin signaling, and fibrinolytic shutdown, culminating in microvascular thrombosis and organ injury. Emerging clinical and translational data suggest that these same modules may reshape stroke biology: NET-rich thrombi are linked to recanalization failure and thrombolysis resistance; systemic endotheliopathy can destabilize the blood–brain barrier and promote no-reflow; and complement–coagulation crosstalk amplifies neurovascular injury. Beyond acute events, chronic microvascular thrombosis and blood–brain barrier leakage allow fibrin(ogen) and coagulation proteases to signal through microglia and protease-activated receptors, potentially coupling vascular dysfunction to cognitive decline and neurodegenerative trajectories. Here we integrate convergent but independently derived evidence from pulmonary medicine, coagulation biology, stroke pathology, and neurodegeneration research into a lung-to-brain immunothrombosis framework. Because these evidence streams have developed largely in parallel, this synthesis represents a mechanistic integration hypothesis—intended to identify shared therapeutic nodes and guide cross-disciplinary validation—rather than a demonstrated biological sequence. We outline biomarker-guided strategies that pair conventional antithrombotics with targeted anti-thromboinflammatory approaches (NET-, complement-, and endothelial/adhesion-directed) while managing bleeding risk.

## 1 Introduction

The lung is a uniquely expansive vascular organ, continuously exposed to external antigens and mechanical stress, and tightly coupled to systemic hemodynamics (Frantzeskaki et al., 2017; Doerschuk, 2001). Pulmonary diseases therefore often co-occur with vascular events, yet the mechanistic depth of this association has been underappreciated (Sá-Sousa et al., 2024; Yeghiazarians et al., 2021; de Bont et al., 2022). Acute respiratory infections and ARDS can precipitate thrombotic complications, while chronic pulmonary disorders and environmental exposures are linked to elevated cardiovascular and cerebrovascular risk over years (Sá-Sousa et al., 2024; Yeghiazarians et al., 2021; de Bont et al., 2022; Bonaventura et al., 2021; Graul et al., 2024). The COVID-19 pandemic made this connection clinically unavoidable by revealing widespread microvascular thrombosis, endotheliopathy, and platelet–leukocyte activation in a primarily respiratory illness, alongside increased rates of thrombotic events and persistent cognitive symptoms in some patients (Bonaventura et al., 2021; Varga et al., 2020; Catherine et al., 2023; Leng et al., 2023).

Traditional explanations for lung-associated stroke and cognitive impairment often focus on hypoxemia, shared risk factors (age, smoking, metabolic disease), and accelerated atherosclerosis (Yeghiazarians et al., 2021; Dahe et al., 2020; Zhang et al., 2023). These mechanisms are important but incomplete: they do not readily account for the microvascular distribution of injury, the inflammatory composition of thrombi, the variability of hemorrhagic complications with thrombolysis, or the persistence of neurocognitive symptoms after apparent pulmonary recovery (Jolugbo and Ariëns, 2021; Staessens et al., 2020; Wang et al., 2021; Jaquet et al., 2022; Hartung et al., 2022). Immunothrombosis and its pathological extension, thromboinflammation, provide a unifying framework that connects innate immune activation to coagulation and microvascular dysfunction (Engelmann and Massberg, 2013; Schrottmaier and Assinger, 2024). In this view, pulmonary injury initiates a coordinated cellular and molecular program involving endothelial activation, platelet immune signaling, NET release, tissue factor-driven thrombin generation, complement amplification, and impaired fibrinolysis, which together shape systemic vascular vulnerability and clot biology (Mandel et al., 2022; Rawish et al., 2021).

**FIGURE 1:**
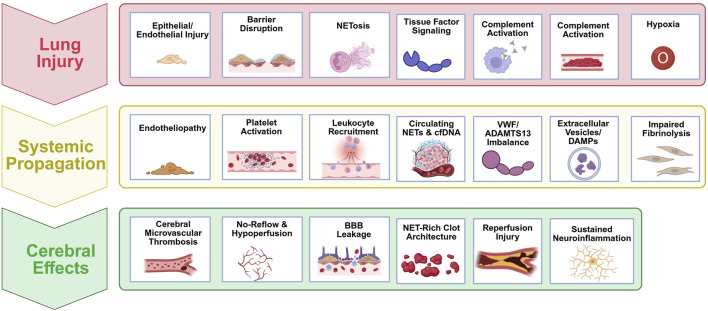
Lung-to-brain immunothrombosis pathway. Pulmonary injury triggers local thromboinflammation, which propagates systemically through endotheliopathy, platelet–leukocyte activation, NETs, VWF/ADAMTS13 imbalance, complement activation, and impaired fibrinolysis. These converge on the cerebral microvasculature to promote microvascular thrombosis, BBB disruption, neuroinflammation, and cognitive decline.

In this review, we first define immunothrombosis and delineate its components and biomarkers. We then examine how major pulmonary disease categories converge on systemic immunothrombosis, emphasizing shared triggers and disease-specific nuances. Next, we synthesize evidence for lung-to-brain transmission mechanisms that may influence stroke pathogenesis and outcomes, and extend the discussion to chronic neurovascular injury and neurodegeneration. Finally, we outline a translational roadmap for biomarker-guided, mechanism-aligned therapies that aim to reduce thrombotic risk while preserving hemostatic safety (Figure 1). Importantly, no single study has yet traced the complete causal chain from a defined pulmonary insult through systemic immunothrombosis to a documented cerebral microvascular event in the same cohort or model. The framework presented here therefore represents a mechanistic integration hypothesis, assembled from convergent but independently derived lines of evidence, intended to guide future validation studies rather than to assert an established pathway.

## 2 Conceptual framework: immunothrombosis and thromboinflammation

Immunothrombosis is the deployment of coagulation as an intravascular effector arm of innate immunity (Engelmann and Massberg, 2013; Schrottmaier and Assinger, 2024). Local microthrombi can restrict pathogen spread and concentrate antimicrobial mediators; when exaggerated or disseminated, the same program can cause endothelial injury, microvascular occlusion, and organ dysfunction (Engelmann and Massberg, 2013; Stark and Massberg, 2021). Thromboinflammation emphasizes reciprocal amplification: inflammatory signaling increases coagulation potential, while thrombin and fibrin feed back on leukocytes and endothelium (Schrottmaier and Assinger, 2024; Stark and Massberg, 2021). Immunothrombosis can be compartmentalized and may precede overt disseminated intravascular coagulation (DIC) in critical illness, underscoring that local microthrombosis can occur without global factor consumption (Gando and Otomo, 2015; Iba et al., 2023).

Endothelial cells both sense and amplify thromboinflammation through barrier disruption, glycocalyx shedding, and a proadhesive phenotype that recruits leukocytes and platelets (Neubauer and Zieger, 2022; Theofilis et al., 2021). Weibel-Palade body release of von Willebrand factor (VWF) promotes platelet tethering; relative reductions in ADAMTS13 activity can permit accumulation of ultralarge VWF multimers and favor platelet-rich microthrombi (Chen and Chung, 2018; Schwameis et al., 2015).

Platelets are key immune effectors in thromboinflammation (Mandel et al., 2022). Activated platelets express P-selectin, release chemokines and procoagulant microparticles, and form platelet–leukocyte aggregates that enhance leukocyte recruitment and tissue factor activity (Mandel et al., 2022; Rohlfing et al., 2021). Platelet innate immune signaling can trigger NET release in septic blood, and platelet hyperreactivity has been described in viral pneumonias including COVID-19, consistent with a primed thromboinflammatory state (Rohlfing et al., 2021; Clark et al., 2007; Yang et al., 2023).

NETs are extracellular chromatin webs released by activated neutrophils (Brinkmann et al., 2004; Fuchs et al., 2007). NETosis can be initiated by pathogen signals, immune complexes, platelet interactions, and hypoxia, and depends on pathways including PAD4-mediated histone citrullination (Papayannopoulos et al., 2010; Li et al., 2010; Thiam et al., 2020a). NETs scaffold platelets and erythrocytes, concentrate tissue factor and complement components, and increase clot rigidity and resistance to fibrinolysis, shaping an “immunothrombotic” clot phenotype (Middleton et al., 2020; Skendros et al., 2020; Gould et al., 2015; Jiménez-Alcázar et al., 2017).

Monocytes and macrophages contribute to immunothrombosis through tissue factor expression, cytokine production, and inflammasome activation (Sachetto and Mackman, 2023; Wu et al., 2021). Complement activation (C3a/C5a and membrane attack complex signaling) intersects with coagulation by activating endothelium and platelets, modulating neutrophils, and altering fibrinolysis; in severe inflammation, PAI-1-associated fibrinolytic shutdown can stabilize microthrombi (Rawish et al., 2021; Dobó et al., 2024; Gando, 2013). These modules are interdependent, enabling broad downstream effects from single-node interventions but also imposing host-defense and hemostatic trade-offs (Rawish et al., 2021).

Fibrinolysis is a key determinant of whether immunothrombosis remains localized or becomes organ-injuring (Gando, 2013). Extracellular DNA and histones can promote clot formation while impairing fibrinolysis and injuring endothelium; NETs further increase resistance to plasmin-mediated lysis (Gould et al., 2015; Jiménez-Alcázar et al., 2017). COVID-19 intensive care unit (ICU) cohorts describe a PAI-1-linked fibrinolysis shutdown phenotype, while DNase and plasmin pathways contribute to NET clearance, suggesting coupling between fibrinolysis competence and NET turnover (Creel-Bulos et al., 2021; Khan, 2021; Demkow, 2023; Meizoso et al., 2021). Operationally, integrated signatures of NET burden, endotheliopathy, complement activation, and impaired fibrinolysis may better capture an actionable immunothrombotic phenotype than single conventional markers (Rawish et al., 2021; Xu et al., 2022a; Natorska et al., 2023; Sinkovits et al., 2022).

**FIGURE 2:**
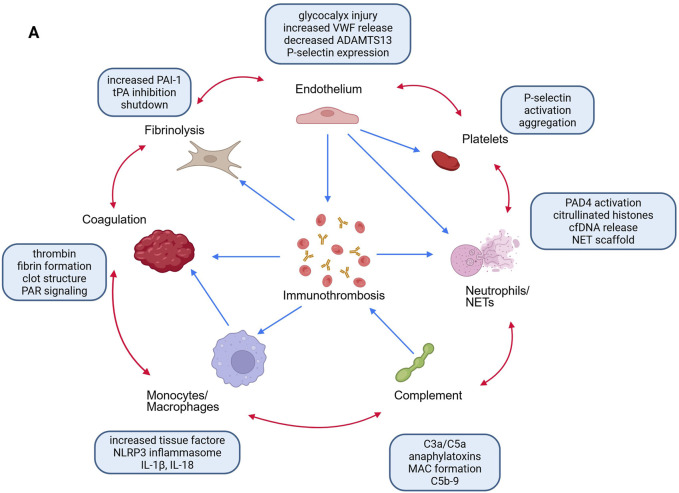
Immunothrombosis network and therapeutic entry points. Bidirectional crosstalk among endothelium, platelets, neutrophils/NETs, complement, monocytes, coagulation, and fibrinolysis creates self-amplifying loops. Labeled tags indicate candidate intervention targets that may be selected by biomarker-defined endotypes.

Translationally, the challenge is to operationalize immunothrombosis as a measurable phenotype (Xu et al., 2022a; Natorska et al., 2023). Candidate panels integrate coagulation activation (e.g., D-dimer and thrombin generation), endothelial injury (e.g., VWF, soluble thrombomodulin, glycocalyx shedding), platelet activation (e.g., soluble P-selectin, platelet–leukocyte aggregates), NET burden (e.g., MPO-DNA complexes, citrullinated histone H3, and cell-free DNA), complement activation (e.g., C5a and soluble C5b-9), and fibrinolytic capacity (e.g., PAI-1) (Chen and Chung, 2018; Schwameis et al., 2015; Dobó et al., 2024; Xu et al., 2022a; Sinkovits et al., 2022; Urano et al., 2019). Because these modules are partially correlated but not redundant, composite endotypes may outperform single biomarkers for risk prediction and for selecting mechanism-matched therapies, and the dominant module may shift with disease phase and vascular bed (pulmonary versus cerebral microcirculation) (Figure 2) (Natorska et al., 2023).

Conceptually, these modules can be organized in a functional hierarchy relevant to the lung-to-brain axis proposed in this review. Upstream initiators—including endothelial activation, tissue factor expression triggered by DAMPs/PAMPs, and hypoxia-driven cellular priming—set the stage for intravascular immune-coagulation engagement. Core amplification loops—platelet–neutrophil cooperation leading to NETosis, complement–coagulation crosstalk, and PAI-1-mediated fibrinolytic shutdown—then expand and stabilize the immunothrombotic response beyond the initial trigger site. Downstream, these amplified signals converge on the cerebral microvasculature, where they may manifest as altered clot composition and microvascular occlusion, impaired reperfusion and thrombolysis resistance, and post-ischemic BBB disruption with secondary neuroinflammation (discussed in Section 4). This hierarchy is not strictly linear—modules feed back on each other extensively, as noted above—but distinguishing initiation, amplification, and downstream brain injury stages provides a useful organizational framework for both mechanistic analysis and therapeutic targeting.

## 3 Pulmonary disease as an upstream driver of systemic immunothrombosis

Pulmonary diseases may initiate immunothrombosis through recurring upstream motifs: epithelial and endothelial injury, hypoxia and shear stress perturbations, pathogen- and damage-associated molecular patterns (PAMPs/DAMPs), and systemic inflammatory spillover (Frantzeskaki et al., 2017; Cardenas et al., 2023). The pulmonary microvasculature is a major site of leukocyte margination and platelet transit, positioning it as a physical and immunologic “filter” where inflammatory signals can be translated into intravascular coagulation (Doerschuk, 2001; Cardenas et al., 2023). Once established in the lung, thromboinflammatory programs can disseminate via circulating cytokines, extracellular vesicles, activated platelets, primed myeloid cells, and soluble coagulation/complement factors, collectively producing systemic endotheliopathy and a hypercoagulable state (Cardenas et al., 2023).

Systemic propagation from lung to distant vascular beds likely involves soluble mediators (cytokines/chemokines), procoagulant extracellular vesicles, and reprogrammed myeloid cells with heightened NET propensity (Gong et al., 2024; Swann et al., 2024; Kalafati et al., 2023). Together, these mediators can sustain endotheliopathy beyond the pulmonary insult, increasing the probability of cerebral microvascular events or worsening outcomes when stroke occurs (Cardenas et al., 2023; Kalafati et al., 2023).

Pulmonary inflammation can also reprogram immunity at the level of the bone marrow (Swann et al., 2024; Kalafati et al., 2023). Emergency myelopoiesis and metabolic reprogramming can yield neutrophils and monocytes with altered effector functions and a lower threshold for NET formation, potentially persisting beyond the acute pulmonary insult (Swann et al., 2024; Kalafati et al., 2023). Hypoxia–reoxygenation and sympathetic activation (e.g., in sleep-disordered breathing or severe pneumonia) may further prime platelets and endothelium, sustaining a systemic thromboinflammatory set point even as respiratory symptoms improve (Swann et al., 2024). These longer-lived changes provide a biologically plausible bridge to delayed cerebrovascular events and post-acute neurocognitive phenotypes and motivate longitudinal biomarker sampling (Pandharipande et al., 2013).

The evidence base for immunothrombotic mechanisms varies substantially across these categories: acute infectious triggers (particularly COVID-19) are supported by detailed mechanistic and clinical data, whereas chronic conditions (COPD, ILD, OSA, air pollution) rely more on epidemiological associations, surrogate biomarkers, and indirect mechanistic reasoning. Furthermore, the expected clinical phenotypes may differ: acute pulmonary events are more closely linked to short-term stroke triggering and altered thrombus composition (including large-vessel occlusion with NET-rich, treatment-resistant clots), while chronic lung diseases may relate more to progressive small-vessel/microthrombotic injury and cognitive trajectories—although exacerbation windows in chronic diseases (e.g., COPD) may also produce acute-phase stroke phenotypes. These distinctions inform the interpretation of disease-specific evidence presented below.

### 3.1 Acute infections and ARDS (including COVID-19)

In COVID-19, endothelial injury, endotheliitis, and microthrombi are common in the pulmonary microcirculation and other vascular beds (Bonaventura et al., 2021; Varga et al., 2020; Lorini et al., 2021; Kamel et al., 2020). Lung-centric immunothrombosis helps explain severe hypoxemia with high thrombotic risk, and pulmonary microthrombosis can amplify systemic inflammation through ventilation–perfusion mismatch and hypoxic signaling (Bonaventura et al., 2021; Kamel et al., 2020; Poor, 2021).

NET formation is a prominent feature of severe COVID-19 and has been directly linked to immunothrombosis in COVID-19 ARDS (Middleton et al., 2020; Cesta et al., 2023). Mechanistically, NETs can present tissue factor, bind VWF, and activate complement, while platelet–neutrophil interactions enhance NET release and local thrombin generation (Middleton et al., 2020; Skendros et al., 2020; Yang et al., 2020). Complement and tissue factor-enriched NETs have been proposed as key drivers of COVID-19 immunothrombosis, consistent with a model in which innate immune amplification is inseparable from coagulation activation (Skendros et al., 2020; Cesta et al., 2023).

Platelet and megakaryocyte responses further promote COVID-19 thromboinflammation (Rohlfing et al., 2021; Zhu et al., 2022; Portier et al., 2021). Platelet hyperreactivity and platelet–leukocyte aggregates can reinforce NETosis and clot stability, and pulmonary vascular injury may manifest as *in situ* thrombosis in addition to thromboembolism (Rohlfing et al., 2021; Poor, 2021; Portier et al., 2021). These programs may affect brain outcomes by reshaping clot architecture and microvascular perfusion (Catherine et al., 2023; Portier et al., 2021).

Markers of endotheliopathy and VWF/ADAMTS13 imbalance are commonly reported in hospitalized COVID-19 cohorts and have been associated with severity and mortality (Sinkovits et al., 2022; Seth et al., 2022; Xu et al., 2022b). A relative reduction in ADAMTS13 activity can promote accumulation of ultralarge VWF multimers, facilitating platelet-rich microthrombi in the microcirculation (Seth et al., 2022; Xu et al., 2022b). Complement activation appears to intersect with this axis, suggesting that combined endotheliopathy–complement phenotypes may identify high-risk subgroups (Sinkovits et al., 2022). Multi-omic studies further support the concept that severe COVID-19 represents a systemic vascular disease with layered immune and coagulation dysregulation (Filbin, 2022; Overmyer et al., 2021).

Non-COVID ARDS and severe pneumonia share similar upstream triggers (barrier loss, leukocyte activation, dysregulated coagulation) within the pulmonary microvasculature (Frantzeskaki et al., 2017; Leung and Middleton, 2024). Fibrinolysis shutdown in critical illness may help sustain microvascular obstruction and prolong systemic thromboinflammatory tone (Gando, 2013; Levy and Iba, 2024).

In sepsis, pneumonia, and trauma, immunothrombosis can progress toward sepsis-induced coagulopathy and DIC in subsets (Gando and Otomo, 2015; Iba et al., 2023; Gando et al., 2025; Semeraro et al., 2015). This spectrum matters because systemic coagulation activation can coexist with regional microthrombosis and organ-specific vulnerability, including the brain, creating a temporally constrained post-insult vascular risk window (Iba et al., 2023; Gando et al., 2025).

A caveat is warranted regarding generalizability. Much of the mechanistic evidence for pulmonary immunothrombosis derives from COVID-19, in which SARS-CoV-2 can directly infect endothelial cells via ACE2, producing endotheliitis that may not occur in other pulmonary triggers such as COPD, ILD, or air pollution exposure (Bonaventura et al., 2021; Varga et al., 2020). While the downstream thromboinflammatory modules (NETs, complement, VWF/ADAMTS13 imbalance) are shared across conditions, the intensity and kinetics of their activation likely differ when direct viral endothelial tropism is absent. Extrapolation from COVID-19 to other pulmonary diseases should therefore be made cautiously, and disease-specific validation of each mechanistic node is needed.

In summary, acute pulmonary infections and ARDS engage the full spectrum of immunothrombotic modules—endotheliopathy, NETosis, complement amplification, and fibrinolytic shutdown—with the most robust evidence from COVID-19. Candidate therapeutic targets in this setting include NET-scaffold disruption (e.g., DNase), complement blockade, and biomarker-guided anticoagulation intensity.

### 3.2 COPD, chronic bronchitis, and smoking-related inflammation

COPD is characterized by chronic airway inflammation and systemic spillover that promote endothelial dysfunction and a prothrombotic milieu (Mkorombindo and Dransfield, 2021; Rahaghi and Pistenmaa, 2021; Simons et al., 2024). Platelet hyperreactivity and impaired fibrinolysis have been reported in stable disease and exacerbations, and exacerbations may create transient “risk windows” for acute cardiovascular and cerebrovascular events (Graul et al., 2024; Simons et al., 2024; Mallah et al., 2020).

Cardiovascular comorbidity is common in COPD and contributes substantially to adverse outcomes (Sá-Sousa et al., 2024; Dahe et al., 2020). Systematic reviews emphasize elevated cardiovascular risk, and population-based studies suggest that exacerbations are followed by a period of heightened risk for nonfatal cardiovascular events (Graul et al., 2024; Müllerová et al., 2022). Whether routine intensification of antithrombotic therapy is beneficial remains uncertain and must be weighed against bleeding risk in older patients with a high comorbidity burden (Petris et al., 2021).

Mechanistically, COPD may amplify immunothrombosis through sustained neutrophil priming and NET-related pathways (Uddin et al., 2019; Keir and Chalmers, 2022). NET-derived DNA can engage innate immune sensors (e.g., cGAS/TLR9) and promote autoimmunity-associated inflammatory signaling in COPD, offering a plausible bridge from chronic airway inflammation to systemic immune activation and coagulation phenotypes (Keir and Chalmers, 2022; Chen et al., 2024).

In summary, COPD engages immunothrombosis through endothelial dysfunction, platelet hyperreactivity, sustained neutrophil priming, and impaired fibrinolysis, with exacerbations creating transient windows of heightened cerebrovascular risk. The most directly aligned interventions are optimized antithrombotic therapy during and after exacerbations and, speculatively, NET-targeted approaches pending clinical validation.

### 3.3 Interstitial lung disease and pulmonary fibrosis

Fibrotic interstitial lung diseases feature persistent tissue injury, aberrant repair, and vascular remodeling, often accompanied by chronic hypoxia (Fließer et al., 2024; Cenerini et al., 2024). These conditions can promote systemic inflammation and endothelial activation, potentially favoring immunothrombotic phenotypes through sustained DAMP release and altered pulmonary hemodynamics (Fließer et al., 2024; Cenerini et al., 2024). Emerging data link lung fibrosis to increased endothelial activation and vascular barrier dysfunction, consistent with a prothrombotic microenvironment (Fließer et al., 2024; Fainberg et al., 2024). Narrative reviews suggest clinically meaningful links between pulmonary fibrosis and thrombotic pathology, although causality, timing, and optimal antithrombotic strategies remain areas of uncertainty (Cenerini et al., 2024; Khan et al., 2021).

Mechanistically, sustained release of DAMPs (including HMGB1 and S100 proteins) from injured alveolar epithelium can activate monocyte tissue factor expression and prime neutrophils for NETosis independently of viral infection, providing a non-COVID pathway through which fibrotic lung disease may engage immunothrombotic programs. However, direct measurement of NET burden and complement activation in ILD cohorts remains limited, and prospective studies linking these biomarkers to cerebrovascular outcomes in fibrosis patients are lacking.

In summary, the dominant immunothrombotic module in ILD appears to be chronic DAMP-driven innate immune activation (via TLR and inflammasome pathways) coupled with progressive vascular remodeling, but the evidence remains largely observational. Prospective studies incorporating immunothrombotic biomarker panels are needed before specific intervention strategies can be considered.

### 3.4 Obstructive sleep apnea and intermittent hypoxia

Obstructive sleep apnea (OSA) is characterized by recurrent upper-airway obstruction and intermittent hypoxia, which trigger sympathetic activation, oxidative stress, and endothelial dysfunction (Yeghiazarians et al., 2021; Arnaud et al., 2020). OSA is strongly linked to cardiovascular disease and is increasingly recognized as a modifiable contributor to stroke risk and post-stroke recovery (Yeghiazarians et al., 2021; Dharmakulaseelan and Boulos, 2024). Intermittent hypoxia can enhance platelet activation and leukocyte-endothelial interactions, providing a plausible mechanistic route to thromboinflammatory vascular vulnerability (Arnaud et al., 2020; Redline et al., 2023). OSA is also associated with cognitive impairment and altered sleep architecture, suggesting that neurovascular and neuroinflammatory pathways may be engaged chronically (Dharmakulaseelan and Boulos, 2024; Pase et al., 2023).

Intermittent hypoxia is a particularly relevant bridge from pulmonary physiology to coagulation because hypoxia-inducible programs can increase endothelial activation, promote platelet reactivity, and prime neutrophils toward NET formation (Martinez-Garcia et al., 2023; Javaheri et al., 2024). The concept of “hypoxic burden” may better capture vascular risk than traditional summary indices, supporting more mechanistic phenotyping in both epidemiology and trials (Martinez-Garcia et al., 2023). In clinical practice, OSA is highly prevalent after stroke, and adherence to positive airway pressure therapy may modify recovery and long-term vascular risk, although definitive outcome trials remain challenging (Yeghiazarians et al., 2021; Dharmakulaseelan and Boulos, 2024; Gleeson and McNicholas, 2022).

In summary, OSA engages immunothrombosis primarily through the endothelial activation–platelet axis driven by intermittent hypoxia–reoxygenation, with emerging evidence linking OSA severity to cerebral small vessel disease. CPAP therapy and vascular risk factor management represent the most directly aligned upstream interventions, but whether they modify immunothrombotic biomarkers and downstream neurovascular risk requires confirmation in adequately powered trials with neurovascular imaging endpoints.

### 3.5 Air pollution and particulate matter exposure

Inhaled particulate matter can induce pulmonary oxidative stress and inflammation that extend systemically through cytokine spillover, autonomic imbalance, and endothelial activation (Bhatnagar, 2022; Arias-Pérez et al., 2020). Epidemiological evidence links air pollution to cardiovascular morbidity and mortality, and mechanistic reviews highlight pathways involving endothelial dysfunction, platelet activation, and coagulation perturbation (de Bont et al., 2022; Bhatnagar, 2022; Arias-Pérez et al., 2020). In dementia epidemiology, vascular dysfunction has been proposed as a mediator linking fine particulate matter (PM2.5) to cognitive decline; however, mediation analyses have yielded mixed results (Zhang et al., 2023). Large cohort studies report strong associations of hypertension and stroke with incident dementia but limited evidence that these events mediate the PM2.5–dementia relationship (Zhang et al., 2023). Notably, Zhang et al. found no significant association between PM2.5 and incident dementia (HR 1.04; 95% CI 0.98–1.11) and no significant mediation by stroke or hypertension in the HRS cohort, suggesting that immunothrombosis-mediated vascular pathways may not fully account for pollution-related cognitive risk, or that current epidemiological designs lack the resolution to capture these mechanisms. These observations support viewing particulate pollution as an exogenous “pulmonary trigger” capable of initiating systemic thromboinflammatory programs relevant to brain health, although the strength of this link at the population level remains to be established (de Bont et al., 2022; Zhang et al., 2023; Bhatnagar, 2022).

At the population level, both chronic exposure and short-term pollution spikes have been associated with cardiovascular outcomes, implying that thromboinflammatory mechanisms may operate on multiple timescales (de Bont et al., 2022; Hayes et al., 2020). This is conceptually relevant to stroke, where transient shifts in vascular tone, platelet activation, and endothelial function could trigger events in predisposed individuals, while chronic exposure may accelerate vascular aging and small vessel disease (Bhatnagar, 2022; Hayes et al., 2020). Integrating exposure metrics with biomarker and imaging endotypes may help connect environmental pulmonary triggers to individual-level neurovascular risk (Table 1) (de Bont et al., 2022; Bhatnagar, 2022).

**TABLE 1:** Immunothrombotic features across major pulmonary disease triggers.

Pulmonary trigger	Shared upstream drivers	Dominant immunothrombotic modules	Representative biomarkers/phenotypes	Brain-relevant implications	Key references
COVID-19/viral pneumonia/ARDS	Epithelial-endothelial injury, hypoxia, systemic cytokine spillover	Endotheliitis; NETs; platelet–leukocyte aggregates; complement amplification; VWF/ADAMTS13 imbalance; fibrinolysis shutdown	NET biomarkers (MPO-DNA/CitH3); elevated VWF with relative ADAMTS13 reduction; elevated PAI-1; complement activation markers	Increased risk of macro- and microvascular thrombosis; NET-rich clot architecture; potential thrombolysis resistance and hemorrhagic transformation risk	Bonaventura et al. (2021), Varga et al. (2020), Middleton et al. (2020), Skendros et al. (2020), Creel-Bulos et al. (2021), Seth et al. (2022)
Sepsis/bacterial pneumonia/non-COVID ARDS	PAMPs/DAMPs, endothelial barrier loss, leukocyte activation	Immunothrombosis progressing toward sepsis-induced coagulopathy/DIC in some patients; complement–coagulation crosstalk; impaired fibrinolysis	Coagulation derangements plus endotheliopathy markers; NET burden varies by phenotype	Systemic endotheliopathy may increase cerebral microvascular vulnerability and worsen outcomes if stroke occurs	Frantzeskaki et al. (2017), Iba et al. (2023), Gando et al. (2025), Semeraro et al. (2015)
COPD/smoking-related inflammation	Chronic airway inflammation, oxidative stress, recurrent inflammatory surges (exacerbations)	Platelet hyperreactivity; endothelial dysfunction; procoagulant shift; NET priming in subsets	Hypercoagulability markers; platelet activation markers; exacerbation-linked risk windows	Periods of heightened cardiovascular risk after exacerbations may include cerebrovascular vulnerability; chronic thromboinflammation may contribute to cognitive decline risk	Graul et al. (2024), Mkorombindo and Dransfield (2021), Rahaghi and Pistenmaa (2021), Mallah et al. (2020), Chen et al. (2024)
Pulmonary fibrosis/ILD	Persistent tissue injury, chronic hypoxia, vascular remodeling	Endothelial activation and barrier dysfunction; potential prothrombotic microenvironment	Biomarker patterns may identify molecular endotypes; endothelial activation signatures	Chronic hypoperfusion and systemic inflammation may reinforce cerebral microvascular injury and cognitive vulnerability	Fließer et al. (2024), Cenerini et al. (2024), Fainberg et al. (2024)
OSA/intermittent hypoxia	Recurrent hypoxia-reoxygenation, sympathetic activation, oxidative stress	Endothelial activation; platelet reactivity; leukocyte-endothelial adhesion; possible NET priming	“Hypoxic burden” phenotypes; OSA severity endotypes	OSA links to stroke and small vessel disease markers; cognitive impairment associations in cohorts	Yeghiazarians et al. (2021), Pase et al. (2023), Martinez-Garcia et al. (2023), Lee et al. (2023)
Air pollution/PM2.5 exposure	Pulmonary oxidative stress and inflammation; autonomic imbalance	Endothelial dysfunction; platelet activation; coagulation perturbation	Exposure-linked cardiovascular risk phenotypes; vascular risk factor interactions	Potential contribution to vascular aging and small vessel disease; mediation by vascular events appears mixed across cohorts	de Bont et al. (2022), Zhang et al. (2023), Bhatnagar (2022), Hayes et al. (2020)

In summary, particulate matter exposure may initiate immunothrombosis through monocyte tissue factor expression, ROS-dependent neutrophil priming, and systemic endothelial activation, but population-level evidence for vascular mediation of pollution-related cognitive risk remains inconclusive. Exposure reduction is the primary intervention; whether immunothrombotic biomarker panels can identify high-risk individuals warrants investigation.

## 4 From lung to brain: mechanistic links to stroke

The processes discussed in this section span distinct but interconnected phases of cerebrovascular injury. Section 4.1 addresses the upstream vulnerability phase: how systemic endotheliopathy and BBB compromise create a substrate for cerebral microvascular dysfunction. Section 4.2 examines two temporally linked processes—immunothrombotic clot formation (NET-scaffold assembly, altered thrombus composition) and reperfusion failure (thrombolysis resistance, microvascular no-reflow)—which together determine initial stroke severity and treatment response. Section 4.3 discusses complement–coagulation crosstalk as a downstream amplification mechanism that can extend neurovascular injury beyond the primary ischemic territory. This phased framing highlights that therapeutic opportunities may differ by stage: endothelial stabilization during the vulnerability phase, NET-dismantling and adjunctive fibrinolysis during reperfusion, and complement modulation during post-ischemic amplification.

### 4.1 Systemic endotheliopathy and cerebral microvascular vulnerability

The neurovascular unit and blood–brain barrier (BBB) impose unique constraints on immune and coagulation signaling (Bardehle et al., 2015). Cerebral microvascular endothelium exhibits specialized tight junctions, low basal leukocyte trafficking, and dependence on pericytes and astrocytic endfeet (Bardehle et al., 2015).When systemic inflammation induces endotheliopathy and glycocalyx loss, cerebral vessels may be particularly vulnerable to microthrombosis and barrier breakdown, in part because BBB disruption can convert normally compartmentalized coagulation mediators into neuroinflammatory stimuli (Theofilis et al., 2021; McLarnon, 2021). At the neurovascular interface, coagulation and fibrinolysis function as a tight control point: small perturbations in hemostasis can have outsized effects on neural homeostasis (Bardehle et al., 2015; McLarnon, 2021).

Endothelial activation also shapes leukocyte recruitment to the brain through adhesion molecules (including P-selectin) and alters VWF dynamics (Chen and Chung, 2018; Denorme et al., 2019). In systemic thromboinflammatory states, VWF/ADAMTS13 imbalance can favor platelet-rich microthrombi, and complement can further amplify endothelial and platelet activation (Garred et al., 2021; Chen and Chung, 2018; Schwameis et al., 2015). These processes provide a plausible mechanistic path from lung-triggered systemic endotheliopathy to cerebral microvascular occlusion and BBB dysfunction, particularly in patients with pre-existing small vessel disease or age-related vascular fragility (Garred et al., 2021; Denorme et al., 2019).

Several lung-derived signals can converge on the neurovascular unit without direct pathogen invasion of the brain (Theofilis et al., 2021; Bardehle et al., 2015). Circulating cytokines and DAMPs can upregulate endothelial adhesion molecules and perturb the glycocalyx; activated platelets and platelet-derived vesicles can carry procoagulant surfaces and inflammatory mediators; and primed neutrophils can marginate and release NETs within cerebral microvessels (Mandel et al., 2022; Theofilis et al., 2021; Bardehle et al., 2015). Because cerebral microvessels operate near a threshold where modest perfusion changes can have functional consequences, small increases in microthrombi or leukocyte plugging may translate into hypoperfusion, BBB leakage, and amplified neuroinflammation (Bardehle et al., 2015; McLarnon, 2021). This framing also helps reconcile why neurological symptoms or cognitive sequelae can follow pulmonary injury even without overt large-vessel stroke: microvascular and barrier phenotypes can be clinically subtle yet biologically consequential (Bardehle et al., 2015; McLarnon, 2021).

### 4.2 NETs and immunothrombotic clot architecture in ischemic stroke

Ischemic stroke thrombi are heterogeneous, containing variable proportions of fibrin, platelets, red blood cells, and leukocyte-derived components (Jolugbo and Ariëns, 2021; Heo et al., 2020). Histological analyses of thrombectomy specimens and systematic reviews of thrombus composition suggest that clot architecture influences both mechanical thrombectomy efficacy and pharmacologic thrombolysis response (Jolugbo and Ariëns, 2021; Staessens et al., 2020; Bhambri et al., 2021). NET-rich thrombi may be more resistant to enzymatic lysis and may increase clot stiffness, thereby contributing to recanalization failure or the need for multiple device passes (Jolugbo and Ariëns, 2021; Heo et al., 2020).

Experimental and clinical studies increasingly support a role for NETs in stroke injury (Denorme et al., 2022; Luo et al., 2023). NETs can promote microvascular “no-reflow,” impair revascularization and vascular remodeling after stroke, and intensify cerebral thromboinflammation (Denorme et al., 2022; Kang et al., 2020; Dhanesha et al., 2022). These effects extend beyond the primary occlusive thrombus and may include secondary microthrombi, endothelial damage, and amplified leukocyte recruitment in the ischemic penumbra (Denorme et al., 2022; Luo et al., 2023). Such mechanisms provide a conceptual explanation for why systemic inflammatory states that increase NET propensity could worsen stroke outcomes even when large-vessel recanalization is achieved (Luo et al., 2023; Kang et al., 2020).

**FIGURE 3:**
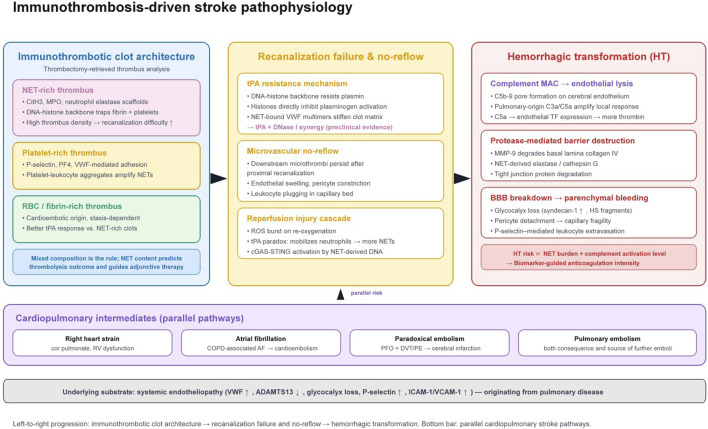
Immunothrombosis-driven stroke pathophysiology. Left: thrombectomy-retrieved clot subtypes (NET-rich, platelet-rich, RBC/fibrin-rich) and their recanalization profiles. Centre: mechanisms of thrombolysis resistance and microvascular no-reflow, including the tPA paradox (tPA-induced NET formation). Right: pathways to hemorrhagic transformation via complement-mediated endothelial lysis, NET-derived protease activity, and glycocalyx loss. Bottom bar: parallel cardiopulmonary stroke pathways. Grey stripe: systemic endotheliopathy as the shared substrate.

NETs also intersect directly with reperfusion therapies (Jolugbo and Ariëns, 2021). The DNA scaffold and associated histones can increase clot stiffness and may reduce the effectiveness of plasmin-mediated fibrinolysis, contributing to thrombolysis resistance and persistent microvascular obstruction (Jolugbo and Ariëns, 2021; Jiménez-Alcázar et al., 2017). This has motivated combined strategies that pair fibrinolysis with NET dismantling (e.g., DNase) to improve clot susceptibility and downstream perfusion (Di et al., 2025). Translationally, studies have begun to relate NET biomarkers measured in blood to NET content within extracted thrombi, supporting the feasibility of biomarker-guided identification of NET-rich clot phenotypes relevant to treatment selection (Figure 3) (Baumann et al., 2024).

An emerging mechanistic highlight is the intersection between NETs and nucleic-acid sensing pathways (Wang et al., 2021; Sun et al., 2023). In mouse models, NETs have been implicated in tissue plasminogen activator (tPA)-associated intracerebral hemorrhage through cGAS-dependent signaling, suggesting that NET-derived DNA can directly amplify neurovascular injury and bleeding risk in the context of thrombolysis (Wang et al., 2021). Platelet innate immune pathways, including STING signaling, can also aggravate thrombotic inflammation in other settings, raising the possibility of a broader platelet–NET–nucleic acid sensing axis relevant to stroke and systemic inflammatory triggers (Yang et al., 2023; Sun et al., 2023).

### 4.3 Complement–coagulation crosstalk in neurovascular injury

Complement activation can contribute to neurovascular injury by activating endothelium and platelets, modulating leukocyte trafficking, and shaping thrombus stability (Rawish et al., 2021; Garred et al., 2021). Complement is also increasingly viewed as therapeutically tractable across a spectrum of diseases, motivating interest in whether complement modulation could attenuate thromboinflammation without the bleeding risks of broad anticoagulation (Garred et al., 2021). In experimental stroke, complement-related interventions can modulate recovery, although the direction and timing of benefit may depend on the complement component targeted and the phase of injury (acute ischemia versus repair) (Garred et al., 2021; Stokowska et al., 2023).

At the mechanistic level, C5a and its receptor C5aR1 can drive microvascular platelet aggregation and inflammatory vascular injury across diverse clinical entities (including aHUS and COVID-19), suggesting a conserved complement–platelet module that may be particularly relevant in thromboinflammatory syndromes (Rawish et al., 2021; Aiello et al., 2022). In pulmonary disease, this module can amplify microangiopathy and endothelial injury; in neurovascular beds, it offers a plausible route to cerebral microvascular occlusion and BBB disruption during systemic inflammation (Rawish et al., 2021; Aiello et al., 2022). Such conserved pathways motivate complement endotyping as part of patient stratification when considering immune-targeted antithrombotic strategies (Garred et al., 2021; Aiello et al., 2022).

In pulmonary disease contexts, complement activation is prominent in severe COVID-19, and complement-targeting trials have reported variable benefits depending on patient selection and disease severity (Vlaar et al., 2022; Carvelli et al. 2022; Hall et al., 2023; Conway and Pryzdial, 2022). While these trials were designed for respiratory outcomes, they provide human evidence that complement blockade can be deployed at scale during a thromboinflammatory crisis, and they highlight the importance of stratifying by immune and coagulation phenotypes (Vlaar et al., 2022; Carvelli et al. 2022; Hall et al., 2023). These insights are directly relevant to designing stroke-adjacent thromboinflammation trials in patients with pulmonary triggers, where the balance between thrombosis suppression and infection risk is critical (Hall et al., 2023; Conway and Pryzdial, 2022).

### 4.4 Cardiopulmonary intermediates

Pulmonary disease can influence stroke risk indirectly through cardiopulmonary intermediates, including right-heart strain, arrhythmias, and venous thromboembolism with paradoxical embolization (Poor, 2021; Fanaroff and Lopes, 2023). These pathways are clinically important, but the focus of this review is the immunothrombosis axis in which pulmonary inflammation and vascular immune activation jointly reshape clot biology and microvascular integrity (Fanaroff and Lopes, 2023). Integrating cardiopulmonary and immunothrombotic mechanisms may ultimately provide the most accurate patient-level models, particularly in complex syndromes such as severe viral pneumonia (e.g., COVID-19) (Table 2) (Poor, 2021; Fanaroff and Lopes, 2023).

**TABLE 2:** Key immunothrombotic modules and evidence levels relevant to the lung–brain axis.

Module	Core mediators	Mechanistic role in lung–brain axis	Evidence base	Therapeutic entry points	Key references
Endothelium/glycocalyx injury	Glycocalyx shedding, adhesion molecules, Weibel-Palade release	Creates proadhesive, procoagulant vascular surface; primes BBB vulnerability	Human cohorts (COVID endotheliopathy) + mechanistic reviews	Endothelial stabilization; glycocalyx protection; anti-adhesion strategies	Bonaventura et al. (2021), Theofilis et al. (2021), Filbin (2022)
VWF/ADAMTS13 imbalance	Ultralarge VWF multimers, reduced ADAMTS13 activity	Promotes platelet-rich microthrombi in inflammatory endotheliopathy	Cohort studies + systematic reviews in COVID	VWF-targeting (analogy to TTP); restoring VWF/ADAMTS13 balance	Seth et al. (2022), Xu et al. (2022b), Pishko et al. (2025), Mancini et al. (2021)
Platelet immune signaling	P-selectin, platelet EVs, platelet–leukocyte aggregates	Initiates and amplifies leukocyte recruitment and NETosis; shapes clot phenotype	Classic mechanistic work + COVID platelet studies	P-selectin blockade; anti-platelet strategies; inhibit platelet innate pathways	Mandel et al. (2022), Rohlfing et al. (2021), Clark et al. (2007), Neri et al. (2020)
Neutrophils/NETosis	PAD4, elastase/MPO, CitH3, cfDNA	Provides thrombus scaffold, increases clot stiffness, promotes no-reflow and BBB injury	Classic NET discovery + COVID ARDS and stroke models	DNase; PAD4 inhibition; disrupt platelet–neutrophil cooperation	Brinkmann et al. (2004), Li et al. (2010), Middleton et al. (2020), Denorme et al. (2022), Di et al. (2025)
Monocyte TF/inflammasome	Tissue factor, NLRP3/IL-1 axis	Drives thrombin generation; amplifies cytokine-driven coagulation	Human samples + mechanistic reviews	IL-1/NLRP3 modulation; TF pathway targeting (context-dependent)	Sachetto and Mackman (2023), Wu et al. (2021)
Complement–coagulation crosstalk	C3a/C5a, C5aR1, MAC	Activates platelets/endothelium; amplifies microvascular thrombosis	Mechanistic and translational reviews; COVID trials	C5a/C5aR1 blockade; lectin pathway inhibition	Garred et al. (2021), Aiello et al. (2022), Vlaar et al. (2022), Carvelli et al. (2022)
Nucleic-acid sensing	cGAS–STING pathways	Links NET DNA to inflammatory vascular injury; may influence hemorrhagic risk	Stroke models + platelet innate immune signaling studies	cGAS–STING modulation; downstream interferon pathway targeting	Wang et al. (2021), Yang et al. (2023), Sun et al. (2023)
Fibrinolysis shutdown	PAI-1/SERPINE1	Stabilizes microthrombi and sustains organ hypoperfusion	ICU cohorts and mechanistic reviews in COVID	PAI-1 pathway modulation; restore fibrinolysis (high safety bar)	Creel-Bulos et al. (2021), Khan (2021), Meizoso et al. (2021)
Coagulation protease signaling (neuro)	Thrombin–PAR signaling	Can act as proinflammatory neuromodulator when BBB is leaky	Reviews + preclinical cognition data	PAR1-targeting strategies (context-dependent)	Festoff and Citron (2019), Festoff and Dockendorff (2021), Zare et al. (2021)

## 5 Immunothrombosis and neurodegeneration: beyond acute stroke

The following section extends the immunothrombosis framework from acute cerebrovascular events to longer-term neurodegenerative processes. This extension is hypothesis-generating: much of the mechanistic evidence derives from non-pulmonary models (genetic Alzheimer’s disease models, cerebral hypoperfusion studies) and is extrapolated to the pulmonary immunothrombosis context on the basis of shared downstream mediators. Direct validation using lung-injury-to-brain-injury models remains an important gap, and the reader should interpret the evidence accordingly.

### 5.1 Microvascular thrombosis, chronic hypoperfusion, and BBB leakage

The evidence reviewed in this section derives predominantly from non-pulmonary models of BBB disruption and neuroinflammation (e.g., genetic Alzheimer’s disease models, cerebral hypoperfusion models). We extrapolate these findings to the pulmonary immunothrombosis context on the basis of shared downstream mediators (fibrin, thrombin, activated myeloid cells), but direct validation using lung-injury-to-brain-injury models is an important gap. Even in the absence of overt stroke, repeated or persistent microvascular thrombosis can contribute to chronic hypoperfusion and BBB dysfunction, creating a substrate for cognitive decline (Bardehle et al., 2015; McLarnon, 2021). BBB leakage enables blood-derived proteins and immune mediators to enter the brain parenchyma, where they can activate microglia and astrocytes and amplify neuroinflammation (McLarnon, 2021; Mendiola et al., 2023). This paradigm reframes coagulation factors not only as vascular risk markers but also as potential drivers of neurodegenerative microenvironments when barrier integrity is compromised (Bardehle et al., 2015; Mendiola et al., 2023).

These processes are likely to interact with cerebral small vessel disease and age-related vascular fragility, where microinfarcts, impaired perfusion reserve, and microbleeds can accumulate over time (Bian et al., 2023). Conceptually, chronic thromboinflammation may provide a vascular substrate on which classic neurodegenerative pathologies progress, and vascular endotypes may help explain heterogeneity in cognitive trajectories (Bian et al., 2023; Khoury and Chapman, 2025). In preclinical models that combine Alzheimer’s disease pathology with cerebral hypoperfusion, anticoagulant interventions have been reported to preserve white matter integrity and remyelination, supporting the idea that coagulation pathways can modulate neurodegenerative phenotypes when vascular compromise is present (McLarnon, 2021).

### 5.2 Fibrin(ogen)–microglia axis and proinflammatory coagulation signaling

Fibrinogen and fibrin can act as proinflammatory signals in the brain (Merlini et al., 2019; Kim et al., 2023). In a genetic Alzheimer’s disease model, fibrinogen has been shown to induce microglia-mediated synaptic spine elimination and cognitive impairment, providing preclinical mechanistic evidence that coagulation proteins can couple vascular dysfunction to neuronal circuitry changes (Merlini et al., 2019). Whether this fibrin–microglia axis operates similarly in the context of pulmonary-triggered systemic immunothrombosis, where BBB disruption may be more diffuse and less genetically driven, has not been tested. Building on this, fibrin-targeting immunotherapies and strategies aimed at limiting fibrin-driven microglial activation have been proposed as disease-modifying approaches for dementia, illustrating the translational potential of focusing on coagulation-derived neuroinflammatory signals rather than only on classic amyloid or tau pathways (Kim et al., 2023; Kantor et al., 2023).

Recent multi-omic profiling studies further support the idea that blood-derived factors can reprogram microglial states in neurodegeneration, providing mechanistic context for why BBB disruption and vascular injury may accelerate cognitive decline (Mendiola et al., 2023). In parallel, development of humanized anti-fibrin monoclonal antibodies highlights an emerging translational pipeline that explicitly targets coagulation-derived neuroinflammatory signals, potentially enabling interventions that are more selective than systemic anticoagulation (Mendiola et al., 2023; Kantor et al., 2023; Kantor et al., 2025).

**FIGURE 4:**
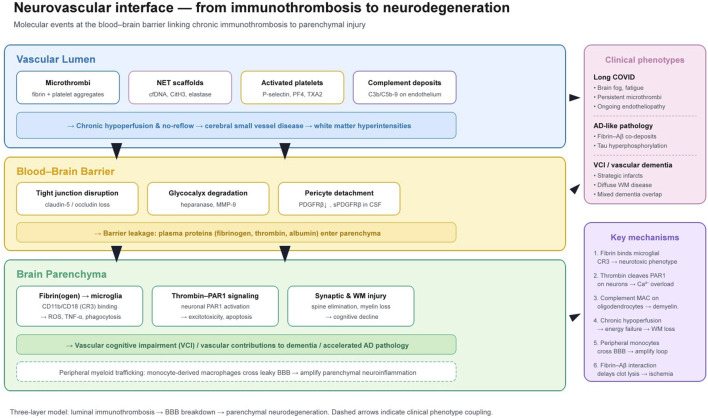
Neurovascular interface linking immunothrombosis to neurodegeneration. Top layer (vascular lumen): microthrombi and NET scaffolds drive chronic hypoperfusion. Middle layer (BBB): tight junction disruption and pericyte detachment allow plasma protein leakage. Bottom layer (parenchyma): extravasated fibrinogen activates microglia (via CR3) and thrombin signals through neuronal PAR1, leading to synaptic loss, white matter injury, and cognitive decline. Right panels: associated clinical phenotypes and key molecular mechanisms.

Beyond fibrin(ogen), thrombin can signal through protease-activated receptors (PARs) to modulate neuroinflammation, synaptic function, and neurotoxicity (Festoff and Citron, 2019; Maoz et al., 2022). Reviews have advanced the concept of “neuro-thromboinflammation” in neurodegenerative disorders and neurotrauma, and preclinical work suggests that PAR1 inhibition may ameliorate cognitive and synaptic plasticity impairments in Alzheimer’s disease models (Festoff and Dockendorff, 2021; Zare et al., 2021). Together, these data motivate a broader view of coagulation proteases as neuromodulatory inflammatory mediators under conditions of BBB compromise (Figure 4) (Festoff and Citron, 2019; Maoz et al., 2022; Festoff and Dockendorff, 2021).

### 5.3 Myeloid trafficking and innate immune memory after lung inflammation

Pulmonary inflammation can imprint peripheral myeloid cells through emergency myelopoiesis and trained immunity-like programs, potentially altering their trafficking and effector function upon subsequent brain injury (Mendiola et al., 2023; Westendorp et al., 2022). After stroke, peripheral immune changes can contribute to infections and influence neurological recovery, while systemic inflammation can worsen neurovascular injury through endothelial activation and microthrombosis (Kalafati et al., 2023). The contribution of lung-primed innate immune memory to neurodegeneration is currently speculative: no study has demonstrated that myeloid cells epigenetically reprogrammed by pulmonary inflammation preferentially traffic to the brain or worsen neurodegenerative outcomes. This remains a high-priority hypothesis for cross-organ systems immunology (Swann et al., 2024; Westendorp et al., 2022).

Bidirectional lung–brain signaling may further reinforce thromboinflammation. Stroke can induce post-stroke immunodepression and pulmonary complications, including infections, which can themselves trigger systemic inflammatory and procoagulant responses. In patients whose stroke occurs on a background of pulmonary disease, these feedback loops may be particularly relevant, because pulmonary reserve is limited and recurrent inflammatory surges can sustain endothelial activation and microvascular dysfunction. Framing these interactions as a coupled lung–immune–brain network may help guide both prevention and rehabilitation-oriented therapeutic strategies (Westendorp et al., 2022; Chen et al., 2025).

### 5.4 Long COVID and post-viral syndromes

Post-acute sequelae of SARS-CoV-2 infection (PASC, or long COVID) include cognitive complaints and neurological symptoms in a subset of individuals (Leng et al., 2023; Talwar et al., 2025). Mechanistic hypotheses include persistent inflammation, endothelial dysfunction, microvascular thrombosis, and autoimmune phenomena, but evidence quality varies and causal inference remains challenging (Leng et al., 2023; Talwar et al., 2025; Shafqat et al., 2023; Afrisham et al., 2022). Given the strong thromboinflammatory signatures observed in acute COVID-19, it is plausible that persistent or relapsing immunothrombosis-related pathways could contribute to neurocognitive symptoms in selected phenotypes, particularly in individuals with pre-existing vascular vulnerability (Shafqat et al., 2023; Afrisham et al., 2022). Clearer endotyping with biomarkers and longitudinal imaging will be essential to move beyond descriptive associations (Talwar et al., 2025).

## 6 Biomarkers, imaging, and patient stratification

A key translational challenge is identifying patients in whom pulmonary disease has induced an actionable immunothrombotic phenotype (Neubauer and Zieger, 2022). Candidate biomarker panels span coagulation activation (e.g., D-dimer, thrombin generation markers), endothelial injury (e.g., VWF, soluble thrombomodulin, glycocalyx shedding markers), platelet activation (e.g., soluble P-selectin, platelet–leukocyte aggregates), NET burden (e.g., MPO-DNA complexes, citrullinated histone H3, cell-free DNA), and complement activation (e.g., C5a, soluble C5b-9) (Neubauer and Zieger, 2022; Sinkovits et al., 2022; Marco and Marco, 2021). In COVID-19, systematic reviews and cohort studies have linked VWF/ADAMTS13 imbalance to disease severity, supporting its use as an endotheliopathy signature that may generalize to other thromboinflammatory contexts (Dobó et al., 2024; Sinkovits et al., 2022; Xu et al., 2022b).

For NET-related biomarkers, increasing clinical work supports both prognostic relevance and biological plausibility (Xu et al., 2022a; Natorska et al., 2023). In cerebrovascular disease cohorts, NET-associated measures and neutrophil and platelet activation signatures have been linked to inflammatory risk and may carry predictive value, and emerging studies directly relate NET biomarkers in blood to NET content in retrieved thrombi (Baumann et al., 2024; Li et al., 2024). These advances support the feasibility of endotyping patients into NET-high versus NET-low phenotypes, which is particularly relevant for selecting adjunctive strategies (e.g., DNase) and for estimating risks such as thrombolysis resistance or hemorrhagic transformation (Natorska et al., 2023; Baumann et al., 2024). Standardization of assays and harmonization across cohorts will be essential for clinical translation (Xu et al., 2022a).

Because immunothrombosis is dynamic, single time-point measurements can misclassify patients (Dobó et al., 2024; Xu et al., 2022a). Serial sampling around key clinical transitions (e.g., escalation of respiratory support, reperfusion therapy, and early recovery) may better capture biomarker trajectories and therapeutic windows. Harmonizing assay platforms and pre-analytical handling is particularly important for NET and complement biomarkers, which are sensitive to sample processing. Embedding standardized panels into multicenter cohorts will be essential for generalizable endotypes (Dobó et al., 2024; Xu et al., 2022a).

Blood biomarkers should be interpreted together with imaging and, when available, clot-level tissue information (Staessens et al., 2020). In thrombectomy-treated stroke, thrombus histology and multi-omic profiling can contextualize NET density, platelet-rich architecture, and fibrin organization, and can be related to recanalization performance and hemorrhagic complications (Jolugbo and Ariëns, 2021; Staessens et al., 2020; Heo et al., 2020; Bhambri et al., 2021; Staessens et al., 2021). Operationally, the goal is to move from static single-marker risk labeling to dynamic multimodal endotyping that guides mechanism-matched, safety-gated therapy intensity over time (Table 3) (Heo et al., 2020).

**TABLE 3:** Selected clinical evidence linking pulmonary triggers to stroke and/or cognitive outcomes.

Pulmonary condition/exposure	Study design/population	Neurovascular/cognitive outcome	Key quantitative findings	Notes/limitations	Key references
COPD exacerbation	Population-based cohort (England primary care-derived COPD cohort, 2014–2020)	Nonfatal hospitalized cardiovascular events (including ischemic stroke) after exacerbations	Composite CV events increased 1–14 days after any exacerbation (aHR = 3.19, 95% CI: 2.71–3.76); highest 1–14 days after severe exacerbation (aHR = 14.5, 95% CI: 12.2–17.3)	Composite outcome includes ischemic stroke; individual stroke effect sizes not detailed in abstract	Graul et al. (2024)
OSA and cerebral small vessel disease	Systematic review and meta-analysis (32 observational studies)	Imaging markers (WMH, lacunes, microbleeds)	Odds of WMH vs. no OSA: mild OR = 1.7 (95% CI: 0.9–3.6), moderate-to-severe OR = 3.9 (95% CI: 2.7–5.5), severe OR = 4.3 (95% CI: 1.9–9.6); moderate-to-severe OSA associated with higher lacunar infarct risk	Unadjusted ORs; heterogeneity across imaging protocols	Lee et al. (2023)
OSA and cognition	Pooled analysis across 5 population cohorts with PSG (n = 5,946; no stroke/dementia)	Global cognition over 0–5 years	Mild-to-severe OSA (AHI ≥ 5) associated with poorer global cognition (pooled β −0.06; 95% CI -0.11 to −0.01); sleep maintenance efficiency associated with better cognition (β 0.08 per 1% increase; 95% CI: 0.03–0.14)	Observational; cognition not dementia incidence	Pase et al. (2023)
COVID-19 ARDS survivors (intubated)	Prospective ICU survivor follow-up (France; 3–6 months post-ICU)	Functional and cognitive outcomes	Favorable functional outcome (mRS < 2) in 39%; mild cognitive impairment (MoCA < 26) in 52% of tested; 74% required rehabilitation	Small sample; follow-up limited to survivors able to attend clinic	Jaquet et al. (2022)
Post-COVID fatigue and cognition	Prospective multicentre cohort with controls (Germany; n = 969; ≥6 months)	Fatigue and cognitive impairment	Clinically relevant fatigue 19% vs. 8% controls; mild cognitive impairment 26%, moderate 1%; only 5% had both fatigue and cognitive impairment	Symptom-based cohorts; mechanisms likely heterogeneous	Hartung et al. (2022)
PM2.5 and dementia (mediation)	US population-based cohort (HRS; 1998–2016)	Incident dementia; stroke/hypertension as mediators	PM2.5 per IQR not associated with dementia (HR = 1.04; 95% CI: 0.98–1.11); prevalent stroke associated with dementia (HR = 1.67; 95% CI: 1.48–1.88); no significant mediation by stroke/hypertension	Mixed evidence across studies; other mediators likely contribute	Zhang et al. (2023)

An important distinction should be drawn between biomarkers used for mechanistic insight—which help delineate immunothrombotic pathways in research settings—and those suitable for clinical decision-making, which require standardized assays, established reference ranges, and validated actionable thresholds. Most immunothrombosis biomarkers discussed above (e.g., MPO-DNA complexes, CitH3, sC5b-9) currently fall into the former category: they have demonstrated prognostic associations in cohort studies but lack the assay harmonization and prospective clinical trial validation needed for point-of-care use. Key steps toward clinical implementation include: (i) multicenter harmonization of sample collection, processing, and storage protocols, particularly for NET and complement markers that are sensitive to pre-analytical variables; (ii) establishment of clinically meaningful thresholds through receiver operating characteristic analysis in prospective cohorts with cerebrovascular endpoints; (iii) demonstration that biomarker-guided treatment selection improves outcomes compared with empirical management in adequately powered trials; and (iv) development of rapid-turnaround assay platforms compatible with acute-care decision timelines. Until these steps are completed, the proposed endotyping framework should be regarded as a research tool that may inform future clinical stratification rather than a ready-to-deploy clinical algorithm.

## 7 Therapeutic implications

### 7.1 Conventional antithrombotic therapy

Conventional antiplatelet and anticoagulant therapies remain foundational for stroke prevention and venous thromboembolism prophylaxis in hospitalized pulmonary patients (Leentjens et al., 2021). In COVID-19, trials comparing prophylactic versus therapeutic-dose heparin show severity- and timing-dependent benefit with nontrivial bleeding risk, highlighting the core trade-off: suppressing microvascular thrombosis while avoiding hemorrhagic complications, including in patients with blood–brain barrier vulnerability or thrombolysis exposure (Lawler et al., 2021; Goligher et al., 2021; Baumann Kreuziger et al., 2022).

Beyond COVID-19, optimal antithrombotic intensity likely depends on mechanism (Wang et al., 2025). Platelet-rich microthrombi supported by VWF and NET scaffolds may respond differently from erythrocyte-rich thrombi, and endothelial injury may raise both thrombosis and bleeding risk (Jolugbo and Ariëns, 2021; Denorme et al., 2019). Mechanistic endotyping could guide when to escalate conventional therapy versus prioritize adjunctive anti-thromboinflammatory targets (Jolugbo and Ariëns, 2021; Wang et al., 2025).

Stroke-specific safety considerations are central when pulmonary thromboinflammation coincides with reperfusion therapy (Jolugbo and Ariëns, 2021; Baumann et al., 2024). NET-rich clots and endothelial injury may increase thrombolysis resistance while simultaneously raising hemorrhagic transformation risk, especially when blood–brain barrier integrity is compromised (Jolugbo and Ariëns, 2021; Wang et al., 2021). In experimental stroke, NET-derived DNA has been implicated in tPA-associated intracerebral hemorrhage through nucleic-acid sensing pathways, suggesting that adjunctive anti-NET strategies could, in principle, improve both recanalization and safety (Wang et al., 2021; Di et al., 2025). Translating this concept will require clinical studies that link biomarker trajectories and thrombus composition to hemorrhagic complications in patients with concurrent pulmonary triggers (Jolugbo and Ariëns, 2021; Baumann et al., 2024).

### 7.2 Targeting NETosis and NET clearance

NET disruption is a mechanism-aligned strategy to reduce thrombus rigidity and microvascular occlusion (Demkow, 2023; Mutua and Gershwin, 2021). Approaches include NET degradation (e.g., DNase), inhibiting NETosis pathways, and blocking platelet–neutrophil cooperation (Li et al., 2010; Demkow, 2023; Mutua and Gershwin, 2021). In stroke models, DNase can reduce ischemic injury without increasing bleeding in preclinical studies and may mitigate tPA-associated hemorrhagic transformation, suggesting a path to widen reperfusion therapy windows (Kang et al., 2020; Di et al., 2025). Translational barriers include timing, infection risk, and biomarkers that identify NET-driven phenotypes (Li et al., 2010; Demkow, 2023; Di et al., 2025).

Target choice within the NET axis matters. PAD4 contributes to antibacterial NET-mediated immunity, raising safety concerns for systemic PAD4 inhibition during active infection; timed NET degradation or downstream pathway targeting (e.g., cGAS–STING signaling, or platelet–neutrophil initiation) may offer safer leverage. Translation will require careful definition of infection status and disease phase (Wang et al., 2021; Yang et al., 2023; Li et al., 2010; Thiam et al., 2020b).

### 7.3 Targeting endothelial activation and adhesion

Because endothelial activation amplifies thromboinflammation, strategies that stabilize endothelium or block adhesion may yield dual anti-inflammatory and antithrombotic effects (Neubauer and Zieger, 2022; Theofilis et al., 2021; Chen and Chung, 2018). P-selectin blockade can reduce platelet–leukocyte interactions and has been explored in COVID-19-related ARDS (Neri et al., 2020). More broadly, modulating VWF release, protecting glycocalyx, or attenuating endothelial inflammatory signaling could reduce microvascular thrombosis and blood–brain barrier vulnerability, but safety windows are critical (Neubauer and Zieger, 2022; Theofilis et al., 2021; Chen and Chung, 2018).

The VWF/ADAMTS13 axis is attractive because it translates endothelial activation into platelet-rich microthrombi (Schwameis et al., 2015). VWF-directed strategies are clinically established in thrombotic thrombocytopenic purpura, but repurposing analogous approaches for pulmonary-triggered endotheliopathy will require evidence of causality and careful bleeding-risk management (Schwameis et al., 2015; Sukumar et al., 2021; Pishko et al., 2025).

### 7.4 Complement and inflammasome modulation

Complement and inflammasome pathways sit at the intersection of innate immune amplification and coagulation (Garred et al., 2021; Potere et al., 2023). Complement-targeting therapies have expanded, and COVID-19 trials provide proof-of-principle that complement blockade can be tested during thromboinflammatory crises (Garred et al., 2021; Vlaar et al., 2022; Carvelli et al. 2022). NLRP3/IL-1 signaling has also been implicated in COVID-19-associated coagulopathy, suggesting that selected anti-inflammatory therapies might dampen coagulation activation; however, infection risk and phenotype selection remain central (Hall et al., 2023; Potere et al., 2023).

Platform-like trials such as the COVID-19 TACTIC-R study illustrate how immune targets (e.g., ravulizumab) can be tested during acute thromboinflammation (Vlaar et al., 2022; Hall et al., 2023). Translating these lessons to pulmonary-triggered stroke risk will require endpoints capturing microvascular function and neurovascular safety, not only systemic thrombotic event rates (Aiello et al., 2022; Hall et al., 2023).

### 7.5 Precision strategy: biomarker-guided combination therapy

**FIGURE 5:**
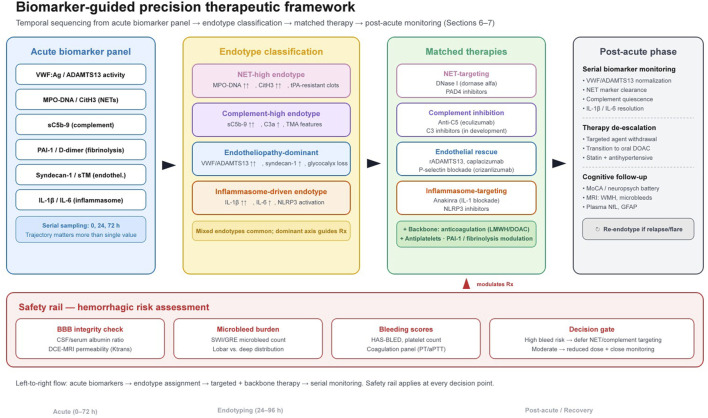
Biomarker-guided precision therapeutic framework (proposed, not clinically validated). Left: a six-marker panel sampled serially (0, 24, 72 h) during the acute phase. Centre: classification into four endotypes (NET-high, complement-high, endotheliopathy-dominant, inflammasome-driven; mixed endotypes acknowledged). Right: endotype-matched targeted therapies layered on a conventional antithrombotic backbone. Far right: post-acute biomarker monitoring, therapy de-escalation, and cognitive follow-up. Bottom safety rail: hemorrhagic risk assessment (BBB integrity, microbleed burden, HAS-BLED) gates all treatment decisions.

A precision strategy is needed given heterogeneity of pulmonary triggers and host vascular vulnerability. A pragmatic approach combines a conventional antithrombotic backbone with a targeted anti-thromboinflammatory agent selected by a biomarker-defined endotype (NET-high, complement-high, or VWF/ADAMTS13-defined endotheliopathy), while explicitly managing hemorrhagic risk (Figure 5) (Wang et al., 2021; Rawish et al., 2021; Schwameis et al., 2015; Demkow, 2023; Matthay et al., 2020).

One testable approach is sequencing: during the most inflammatory phase, emphasize endothelial stabilization and immune-targeted antithrombotic strategies (NET and complement axes) to reduce microvascular thrombosis and preserve barrier integrity; after infection resolves, optimize conventional antithrombotics for longer-term prevention (Chen and Chung, 2018; Demkow, 2023; Vlaar et al., 2022). Biomarker trajectories may help guide switching, for example, persistent VWF/ADAMTS13 imbalance as an endotheliopathy signature versus persistent NET markers indicating ongoing scaffold-driven thrombosis (Chen and Chung, 2018; Demkow, 2023; Vlaar et al., 2022). Adaptive platform trials (including recent COVID-19 thromboinflammation studies) with adaptive enrichment and prespecified hemorrhage safety stopping rules could evaluate such strategies across pulmonary triggers (Table 4) (Vlaar et al., 2022; Hall et al., 2023).

**TABLE 4:** Therapeutic strategy matrix for lung-triggered thromboinflammation and brain outcomes.

Target axis	Example interventions	Evidence base	Potential pulmonary contexts	Brain/stroke-relevant considerations	Biomarkers for stratification	Key references
Conventional antithrombotics	Heparin anticoagulation; antiplatelet therapy; DOACs (context-specific)	RCTs and meta-analyses in COVID; established for stroke prevention	Hospitalized severe infections/ARDS; chronic pulmonary disease with vascular comorbidity	Balance thrombosis reduction vs. intracranial bleeding (especially with thrombolysis/BBB injury)	D-dimer; bleeding risk markers; imaging (microbleeds)	Wang et al. (2025), Lawler et al. (2021), Goligher et al. (2021), Baumann Kreuziger et al. (2022), Ena and Valls (2023)
NET disruption/clearance	DNase; timed NET degradation	Preclinical stroke studies; translational reviews	NET-high inflammatory pneumonias; post-infectious thromboinflammation	May improve microvascular perfusion and thrombolysis responsiveness; infection risk depends on target	MPO-DNA/CitH3; cfDNA; thrombus NET content	Demkow (2023), Di et al. (2025), Baumann et al. (2024), Mutua and Gershwin (2021)
NET formation upstream	PAD4 inhibition; inhibit platelet–neutrophil initiation pathways	Mechanistic studies; safety concerns in infection	Chronic NET-driven inflammation; post-acute phases	PAD4 important for antibacterial defense; timing is critical	NET biomarkers; infection status	Clark et al. (2007), Li et al. (2010), Thiam et al. (2020b)
Complement blockade	Anti-C5a (vilobelimab); anti-C5aR1 (avdoralimab); lectin pathway inhibition	RCTs in severe COVID; complement therapeutics landscape	Severe thromboinflammatory respiratory failure	Potential to reduce microvascular platelet aggregation; monitor infection risk	C5a; sC5b-9; endotheliopathy signatures	Garred et al. (2021), Aiello et al. (2022), Vlaar et al. (2022), Carvelli et al. (2022)
Endothelial/adhesion targeting	P-selectin blockade; endothelial stabilization	Early translational work in ARDS (including COVID-19-related ARDS); mechanistic rationale	ARDS/endotheliopathy phenotypes	May reduce leukocyte recruitment/NET initiation; safety in bleeding-prone patients	Soluble P-selectin; platelet–leukocyte aggregates	Mandel et al. (2022), Neri et al. (2020)
VWF/ADAMTS13 axis	VWF-targeting (analogy to TTP)	Clinical proof-of-principle in TTP; COVID endotheliopathy associations	Endotheliopathy with platelet-rich microthrombi	Potential to reduce microthrombi; must consider intracranial bleeding risk	VWF/ADAMTS13 imbalance	Seth et al. (2022), Xu et al. (2022b), Pishko et al. (2025)
Neuro-thromboinflammation (BBB-leak contexts)	PAR1-targeting (preclinical)	Reviews and animal studies	Chronic vascular injury phenotypes	Targets coagulation-derived neuroinflammatory signaling; translation uncertain	BBB leakage markers; neuroinflammation markers	Festoff and Citron (2019), Festoff and Dockendorff (2021), Zare et al. (2021)

Operationally, the objective is adaptive control of dominant pathway activity rather than maximal suppression of all inflammatory and coagulation signals (Staessens et al., 2020; Bhambri et al., 2021). Platform trials should therefore combine dynamic endotyping with predefined neurovascular safety thresholds and pathway-specific stopping rules (Jolugbo and Ariëns, 2021; Bhambri et al., 2021).

### 7.6 Cross-cutting safety trade-offs

Across the intervention classes discussed above, three mechanistic targets can be distinguished: (a) thrombus structure and NET scaffold (DNase, PAD4 inhibitors, anti-histone strategies), which aim to reduce clot rigidity and microvascular occlusion; (b) the endothelium and adhesion axis (P-selectin blockade, VWF-directed strategies, glycocalyx protection), which target the interface between immune activation and platelet recruitment; and (c) coagulation–inflammation amplification loops (complement inhibitors, inflammasome modulators, anticoagulant dose escalation), which aim to interrupt feed-forward cycles that sustain thromboinflammation. Two cross-cutting safety trade-offs apply to all three categories. First, strategies targeting NETs, complement, and inflammasome pathways inherently modulate host defense; during active infection—the very context in which immunothrombotic modules are most strongly activated—the risk of secondary infection or impaired pathogen clearance must be weighed against antithrombotic benefit, as suggested by the variable outcomes observed in complement blockade trials during active COVID-19 infection. Sustained complement inhibition additionally carries a recognized risk of encapsulated-organism infections (e.g., meningococcal disease), and timing relative to infection resolution is likely critical for all immune-targeted strategies. Second, intensified anticoagulation or combined antithrombotic strategies increase bleeding risk, particularly in patients with blood–brain barrier compromise, recent thrombolysis, or concurrent thrombocytopenia; predefined hemorrhagic safety thresholds are therefore essential in any trial of biomarker-guided combination therapy.

## 8 Discussion

This review differs from prior immunothrombosis syntheses that focus on a single disease context (e.g., COVID-19 coagulopathy or stroke thrombus biology) by tracing a cross-organ pathway from pulmonary triggers through systemic thromboinflammation to cerebral microvascular and neurodegenerative outcomes. The integrative framework is intended to highlight shared mechanistic nodes that may be therapeutically tractable across pulmonary conditions, rather than to imply that all lung diseases engage identical pathways with equal intensity.

Several cross-cutting observations emerge from comparing pulmonary triggers (Table 1). Endothelial activation, platelet hyperreactivity, and impaired fibrinolysis appear to be genuinely shared modules, whereas the contribution of NETs and complement amplification varies by disease context and severity. COVID-19 uniquely involves direct ACE2-mediated endothelial infection, which likely amplifies immunothrombosis beyond what occurs in COPD exacerbations, ILD, or pollution exposure. OSA introduces a distinctive intermittent hypoxia–reoxygenation pattern that may prime thromboinflammation through different kinetics than sustained inflammatory insults. These disease-specific nuances argue against a one-size-fits-all therapeutic approach and reinforce the need for endotype-based stratification.

Several limitations should be acknowledged. First, the evidence base is heavily weighted toward COVID-19; mechanistic data from non-COVID pulmonary triggers remain sparse, and head-to-head comparisons across diseases are lacking. Second, the extension to neurodegeneration relies on extrapolation from genetic Alzheimer’s disease models and cerebral hypoperfusion studies rather than direct lung-to-brain injury models. Third, epidemiological support is mixed: at least one large cohort found no significant mediation of the PM2.5–dementia association by stroke or hypertension. Fourth, this is a narrative review without systematic search or formal quality assessment, introducing potential selection bias. Finally, the proposed biomarker-guided precision framework remains conceptual; NET and complement assays are not yet standardized, and no clinical trial has tested endotype-matched anti-thromboinflammatory therapy in this population.

## 9 Conclusions and future directions

Pulmonary disease should be considered not only a respiratory disorder but also a systemic vascular-inflammatory stressor. Across different pulmonary triggers, a recurrent thromboinflammatory architecture emerges, characterized by endothelial activation with VWF/ADAMTS13 imbalance, platelet hyperreactivity and platelet–leukocyte cooperation, NET-mediated scaffold formation, complement amplification, and impaired fibrinolysis. As summarized in Table 5, however, the relative dominance of these modules differs by disease context: COVID-19/ARDS is most strongly associated with NETs, complement activation, severe endotheliopathy, and fibrinolytic shutdown; COPD is linked more closely to neutrophil priming, platelet hyperreactivity, endothelial dysfunction, and impaired fibrinolysis, particularly during exacerbation windows; ILD is associated with chronic DAMP-driven innate immune activation and vascular remodeling; OSA is dominated by an intermittent hypoxia-driven endothelial–platelet axis; and air pollution is linked to monocyte tissue factor signaling, ROS-neutrophil priming, and endothelial activation. This framework helps explain why lung inflammation is linked to higher stroke risk, more treatment-resistant thrombi, incomplete reperfusion, and persistent neurovascular injury that may promote cognitive decline and neurodegenerative trajectories. However, existing evidence primarily stems from COVID-19 studies, with associations to chronic neurodegenerative diseases largely extrapolated from non-pulmonary models; direct validation of the complete lung–brain causal chain remains a critical gap. The next step is to convert this mechanistic model into precision intervention. Priorities include longitudinal clot and blood multi-omics that connect pulmonary triggers to cerebrovascular outcomes, biomarker trajectories that support endotype-based stratification and adaptive trial designs, and biomarker-guided combination therapies that attenuate thromboinflammation while preserving hemostatic safety.

**TABLE 5:** At-a-glance summary: pulmonary triggers, dominant immunothrombotic modules, stroke phenotypes, and aligned interventions.

Pulmonary trigger	Dominant module(s)	Clinical phase/Stroke phenotype	Aligned Intervention(s)	Evidence tier
COVID-19/ARDS	NETs, complement, endotheliopathy, fibrinolysis shutdown	Acute/large-vessel + microvascular	DNase, complement blockade, guided anticoagulation	Clinical trials + mechanistic
COPD	Neutrophil priming, platelet hyperreactivity, endothelial dysfunction, impaired fibrinolysis	Exacerbation windows/small-vessel	Exacerbation-timed antithrombotics	Cohort + mechanistic (biomarker-level)
ILD	Chronic DAMP burden, vascular remodeling	Chronic/microvascular	Under investigation	Observational + preclinical
OSA	Endothelial–platelet axis (IH-driven)	Chronic nocturnal/small-vessel	CPAP, vascular risk factor management	Epidemiological + imaging + preclinical mechanistic
Air pollution	Monocyte TF, ROS-neutrophil priming, endothelial activation	Chronic + acute spikes/small-vessel	Exposure reduction	Epidemiological + *in vitro*

## Glossary

AD: Alzheimer’s disease
ADAMTS13: a disintegrin and metalloprotease with thrombospondin type 1 motifs 13
AHI: apnea-hypopnea index
aHR: adjusted hazard ratio
aHUS: atypical hemolytic uremic syndrome
ARDS: acute respiratory distress syndrome
BBB: blood–brain barrier
cfDNA: cell-free DNA
cGAS: cyclic GMP-AMP synthase
CitH3: citrullinated histone H3
COPD: chronic obstructive pulmonary disease
CR3: complement receptor 3
CV: cardiovascular
DAMPs: damage-associated molecular patterns
DCE-MRI: dynamic contrast-enhanced magnetic resonance imaging
DIC: disseminated intravascular coagulation
DOACs: direct oral anticoagulants
EVs: extracellular vesicles
GFAP: glial fibrillary acidic protein
GRE: gradient echo
HAS-BLED: Hypertension, Abnormal renal/liver function, Stroke, Bleeding history, Labile INR, Elderly, Drugs/alcohol
HR: hazard ratio
ICUintensive care unitIL-1: intensive care unitIL-1interleukin-1
IL-6: interleukin-6
ILD: interstitial lung disease
IQR: interquartile range
LMWH: low-molecular-weight heparin
MAC: membrane attack complex
MMP-9: matrix metalloproteinase-9
MoCA: Montreal Cognitive Assessment
MPO: myeloperoxidase
mRS: modified Rankin Scale
NETs: neutrophil extracellular traps
NfL: neurofilament light chain
NLRP3: NLR family pyrin domain containing 3
OR: odds ratio
OSA: obstructive sleep apnea
PAD4: peptidylarginine deiminase 4
PAI-1: plasminogen activator inhibitor-1
PAMPs: pathogen-associated molecular patterns
PAR1: protease-activated receptor 1
PASC: post-acute sequelae of SARS-CoV-2 infection
PD: Parkinson’s disease
PF4: platelet factor 4
PFO: patent foramen ovale
PM2.5: fine particulate matter
PSG: polysomnography
RBC: red blood cell
ROS: reactive oxygen species
sC5b-9: soluble C5b-9
STING: stimulator of interferon genes
sTM: soluble thrombomodulin
SWI: susceptibility-weighted imaging
TF: tissue factor
TLR9: Toll-like receptor 9
tPA: tissue plasminogen activator
TTP: thrombotic thrombocytopenic purpura
VCI: vascular cognitive impairment
VWF: von Willebrand factor
WMH: white matter hyperintensities
